# Radiotherapy for Oligometastatic Lung Cancer

**DOI:** 10.3389/fonc.2017.00210

**Published:** 2017-09-19

**Authors:** Derek P. Bergsma, Joseph K. Salama, Deepinder P. Singh, Steven J. Chmura, Michael T. Milano

**Affiliations:** ^1^Department of Radiation Oncology, University of Rochester Medical Center, Rochester, NY, United States; ^2^Department of Radiation Oncology, Duke University Health System, Raleigh, NC, United States; ^3^Department of Radiation and Cellular Oncology, University of Chicago, Chicago, IL, United States

**Keywords:** lung cancer, non-small cell lung cancer, oligometastases, stereotactic body radiotherapy, oligometastatic disease

## Abstract

Non-small cell lung cancer (NSCLC) typically presents at an advanced stage, which is often felt to be incurable, and such patients are usually treated with a palliative approach. Accumulating retrospective and prospective clinical evidence, including a recently completed randomized trial, support the existence of an oligometastatic disease state wherein select individuals with advanced NSCLC may experience historically unprecedented prolonged survival with aggressive local treatments, consisting of radiotherapy and/or surgery, to limited sites of metastatic disease. This is reflected in the most recent AJCC staging subcategorizing metastatic disease into intra-thoracic (M1a), a single extra thoracic site (M1b), and more diffuse metastases (M1c). In the field of radiation oncology, recent technological advances have allowed for the delivery of very high, potentially ablative, doses of radiotherapy to both intra- and extra-cranial disease sites, referred to as stereotactic radiosurgery and stereotactic body radiotherapy (or SABR), in much shorter time periods compared to conventional radiation and with minimal associated toxicity. At the same time, significant improvements in systemic therapy, including platinum-based doublet chemotherapy, molecular agents targeting oncogene-addicted NSCLC, and immunotherapy in the form of checkpoint inhibitors, have led to improved control of micro-metastatic disease and extended survival sparking newfound interest in combining these agents with ablative local therapies to provide additive, and in the case of radiation and immunotherapy, potentially synergistic, effects in order to further improve progression-free and overall survival. Currently, despite the tantalizing potential associated with aggressive local therapy in the setting of oligometastatic NSCLC, well-designed prospective randomized controlled trials sufficiently powered to detect and measure the possible added benefit afforded by this approach are desperately needed.

## Introduction

Although lung cancer incidence and mortality are declining, due in large part to public health smoking cessation efforts, it remains the leading cause of cancer-related mortality both in the United States and worldwide (1). The majority of lung cancer patients have non-small cell lung cancer (NSCLC) and commonly present with metastases involving distant organ sites. Historically, palliative treatment with chemotherapy has been the standard of care for metastatic NSCLC, and outcomes with this approach have been frustratingly dismal, resulting in a median overall survival (OS) of only 8–10 months (2). However, in the past 5 years, clinical trials evaluating the efficacy of targeted therapy with receptor tyrosine kinase inhibitors (TKIs) and immunotherapy with checkpoint inhibitors represent a breakthrough in the care of advanced NSCLC offering improvements in progression-free survival (PFS) and OS (3–5). Despite these advances, long-term PFS remains limited to a relatively small subset of metastatic NSCLC patients.

Radiation therapy, a critical component of curative treatment for non-metastatic NSCLC, has classically been reserved for tumor-related symptom palliation in the metastatic disease setting. The past decade has brought about dramatic improvements in the planning and delivery of radiation treatments due to technical advancements in computing power, diagnostic imaging, and motion management. This has led to the increased use of precisely targeted highly conformal radiation, often in large doses per treatment.

Hypofractionated image-guided radiotherapy (HIGRT), typically referred to as “stereotactic radiosurgery” (SRS) when delivered to an intracranial target in one or more fractions or “stereotactic body radiotherapy” or “stereotactic ablative radiotherapy” (SBRT or SABR) when given to extra-cranial body sites in one or more fractions, has been shown to result in high rates of treated tumor control (6–8) with a favorable toxicity profile and improved convenience (9) when compared to conventionally fractionated external beam radiation (EBRT). Although not yet elucidated entirely, it is postulated that hypofractionated radiotherapy may accomplish tumor killing *via* different biological mechanisms than conventional fractionation, one being the possible infliction of endothelial or vascular damage (10, 11).

The role of radiotherapy, delivered as SBRT or conventionally fractionated EBRT with or without systemic therapy, is rapidly evolving with dramatically increased utilization in the treatment of advanced NSCLC patients with limited sites of metastatic disease termed “oligometastases” (12). Despite these advances, the appropriate selection of oligometastatic patients for curative-intent local treatment, optimal integration of radiotherapy with systemic therapy, and the added long-term benefit such as aggressive treatment approach provides have not been sufficiently clarified.

## The Oligometastatic State

One of the hallmarks of cancer is the development of pioneer cells that are able to release from the primary tumor site and metastasize to regional lymph nodes and/or distant organs *via* the lymphatics, blood stream, or direct extension (13). The risk of subclinical dissemination in solid tumors, even in the setting of apparently localized disease, is variable and dependent on tumor histology, size, grade, stage, genetics, and a host of other factors, many of which are not yet understood. Hellman and Weichselbaum postulated the existence of an oligometastatic state in which tumors develop sites of distant metastasis in a single or limited number of organs as a function of the underlying biology of tumor cells and the unique receptiveness of distant organ sites (“seed and soil”) (14). This concept of oligometastases is derived from the spectrum theory that bridges the gap between the Fisher and Halstedian viewpoints on cancer. Fisher argued many tumors are micro-metastatic from inception even when presenting without clinical/radiographic evidence of distant metastatic disease (15), whereas Halsted postulated orderly spread from the primary tumor into regional lymph nodes and ultimately distant organs (16). In the spectrum theory of cancer, the oligometastatic state may reflect patients with more indolent disease courses that may be cured or rendered disease free for long time intervals with aggressive local treatment of distant metastases. We will further discuss the rationale and review the ever-growing clinical evidence supporting an aggressive treatment approach in select NSCLC patients presenting with oligometastatic disease.

Historically, before the development of systemic therapies with increased efficacy, aggressive metastasis-directed treatments were relied upon to palliate, and occasionally cure, patients with limited metastases (17). In the setting of systemic therapy, which may be able to sterilize micrometastases, control of clinically detectable tumors is perhaps of even more importance. Although prospective clinical trials do not routinely report PFS of individual metastases, observational studies report that the predominant pattern of recurrence in patients with oligometastatic NSCLC treated with first-line systemic therapy appears to be local only (18, 19). This pattern of progression would support the potential PFS benefit of delivering aggressive local therapy to all appreciable metastatic sites, as well as the thoracic primary, if feasible. As unchecked growth of oligometastases may culminate in progressive organ dysfunction eventually leading to death, improved PFS with aggressive local therapy may ultimately result in longer OS. In recent decades, the bulk of published clinical series have included patients with oligometastatic sarcoma, colorectal, or breast cancer (20–22); however, there are an increasing number of single institution studies, the majority retrospective, which report long-term PFS and OS associated with aggressive treatment to all known sites of disease in oligometastatic NSCLC (23).

One of the difficulties in interpreting and applying the available data to predict which patients will benefit from an aggressive treatment approach including local therapies is establishing the appropriate cutoff to define the oligometastatic state. Nearly all published studies of oligometastatic NSCLC have limited inclusion to patients with ≤5 metastases; however, the majority enrolled patients with ≤3 metastases and over half of all patients included in a recent meta-analysis had only a single metastasis (24). The oligometastatic state is believed to be a relatively common presentation of advanced NSCLC; however, its exact incidence is dependent on the cutoff used for its definition. The relative prevalence of oligometastatic disease in advanced NSCLC has been reported to range from 26 to 50% using cutoffs of ≤3–5 metastases (18, 25). The reported rates of oligorecurrence after definitive surgical treatment of NSCLC are even higher, with 33–50% of patients recurring with ≤3 lesions (26, 27). It is important to note that the studies reporting rates of oligometastases are likely subjected to selection bias as patients with more metastatic burden may be less likely to be enrolled on protocols and/or treated at tertiary or quaternary referral centers that often report their large institutional experiences.

In general, patients with fewer metastases tend to have better outcomes than those with a more widespread presentation irrespective of the potential impact of aggressive metastasis-directed local therapy. This has been shown in multiple retrospective studies (25, 27, 28) including a comprehensive analysis of advanced NSCLC patients treated on consecutive Southwest Oncology Group prospective protocols that revealed a significantly longer OS in patients developing a single metastasis (8.7 months) vs multiple metastases in a single organ (6.2 months) or multiple organs (5.1 months) (28). Some argue that oligometastases do not reflect a more indolent biology but rather lead-time bias in which patients are found to have metastatic disease at an earlier point in the natural history of their disease. However, this explanation cannot fully account for the long-term survival of some individuals with oligometastatic disease with up to one-quarter of patients surviving long-term with aggressive treatment to all sites of disease (24, 29). The recent 8th edition of the AJCC staging manual now considers a single extrathoracic metastasis to be M1b vs more widespread extrathoracic M1c disease (30). The M1a substage interestingly includes patients with potentially more metastatic burden than M1b, such as numerous lung metastases and/or malignant pleural effusion(s), as long as it is contained to the thorax.

Another important consideration when defining the oligometastatic state is appropriate patient evaluation/staging. Advancements in modern diagnostic imaging, including more widespread use of brain MRI and FDG-PET/CT, have improved the detection of both intra- and extra-cranial metastatic disease. MRI is superior to CT in staging the brain and may detect the presence of metastases, particularly small lesions, unappreciated by CT (31). The use of PET/CT staging is associated with improved OS, likely due in part to stage migration where patients are bumped into a higher stage category by the detection of otherwise clinically unapparent metastases (32, 33). Based on published studies, approximately 15% of NSCLC patients initially thought to be stage I–III may be upstaged to stage IV with use of PET/CT in addition to contrast CT imaging alone (34, 35). Furthermore, modern imaging may detect widespread metastases in patients thought to have oligometastatic disease, thereby avoiding aggressive metastasis-directed local therapy in those who are unlikely to have a PFS benefit. It is important to consider that the bulk of published studies in oligometastatic NSCLC included patients treated before the routine use of PET/CT for staging (24).

Despite a strong focus on using a strict number of metastases to define oligometastatic disease, other factors including age and performance status, volume of disease, histology, tumor location(s), rate of progression, and genetics may be important in predicting benefit from aggressive local therapy (36). For appropriate clarification of distinct clinical scenarios, oligometastatic disease can be subdivided based on the development of metastases in relation to initial diagnosis and systemic therapy (37). Synchronous or *de novo* oligometastases refers to presentation with a limited number of lesions at initial diagnosis, while oligorecurrence is the metachronous development of new metastases after definitive treatment of initial locoregional thoracic disease. Patients with more widespread presentation experiencing relative disease stability on “mostly effective” systemic therapy aside from a limited number of persistent or recurrent/growing metastases may be referred to as having oligoresistance (or “induced oligometastases”) and oligoprogression disease, respectively. The latter two scenarios are fairly common in the setting of oncogene-addicted NSCLC (those patients with ALK rearrangements or EGFR mutations) and are due predominantly to acquired resistance to treatment with TKIs in progressing/resistant tumor clonogens (38).

Timing does appear to be important and an improved prognosis has been observed in patients presenting with oligorecurrence compared to those with *de novo* oligometastases as evidenced by an individual patient data meta-analysis by Ashworth and colleagues including 757 oligometastatic NSCLC patients treated with ablative treatments to all sites of disease which reported the latter to be associated with a HR of 1.96 (*p* < 0.001) on multivariate analysis (24). It is worth mentioning that a more recent publication did not show worse OS outcomes in synchronous patients if treated with aggressive thoracic therapy (ATT) (39). Additional adverse prognostic factors for OS reported in the meta-analysis by Ashworth (24) included higher thoracic stage and/or mediastinal node positivity, presence of brain metastases, non-adenocarcinoma histology, and non-surgical treatment. Nearly 90% of patients included had a single metastatic lesion and the presence of >1 oligometastatic lesion and/or multiple organ involvement was significantly associated with worse PFS. As alluded to previously, there is evidence to support the premise that larger volume, rather than number, of metastases is more predictive of worse outcome. A retrospective study conducted at MD Anderson Cancer Center including 1,284 patients with advanced NSCLC found that the number of extra-cranial metastases correlated with OS; however, among patients with brain- or lung-only metastases, there was an even stronger association with cumulative metastatic tumor volume (40).

## Definitive Radiotherapy-Based Treatment of Oligometastases

Historically, surgical resection has been the preferred metastasis-directed treatment for patients with limited metastases from NSCLC (41). Surgery has the attributes of being both diagnostic, by providing pathologic confirmation of metastatic disease, and therapeutic, by eliminating tumor and/or alleviating tumor-related symptoms (42, 43). The benefit of aggressive metastasis-directed therapy was first shown in patients with limited brain metastases. Patchell and colleagues (44) performed a phase III randomized controlled trial evaluating the impact of surgical resection added to palliative whole brain radiotherapy (WBRT) in a predominantly NSCLC population of patients with a single brain metastasis reporting resected patients lived significantly longer (40 vs 15 weeks, *p* < 0.01). Following this, a number of studies examined the effect of extra-cranial metastasis-directed therapy, typically consisting of surgery and/or radiotherapy, with nearly two-thirds of patients included in a recent meta-analysis of oligometastatic NSCLC patients managed with surgery as the primary treatment. Although surgery was found to be significantly associated with improved OS, there was a strong potential selection bias favoring outcomes in the surgical group (for example, medical co-morbidities) (24).

Unfortunately, many patients with oligometastases present with one or more unresectable deposits and/or may be poor operative candidates due to advanced age and/or medical co-morbidities. There is interest in utilizing less invasive, potentially ablative techniques to treat oligometastases including thermal, cryo-, chemical, or irreversible electroporation ablation, but experience with these techniques is limited and restricted to select institutions (45). Furthermore, their role in the treatment of oligometastatic disease, NSCLC, or otherwise is not well defined.

The ability of modern radiotherapy techniques to deliver potentially ablative HIGRT doses, including SRS and SBRT, to numerous organ sites throughout the body has allowed for the aggressive treatment of unresectable metastases. Techniques intrinsic to SRS were initially developed to treat small targets in the brain that were not amenable to conventional surgery (46), however, have since been greatly refined due to improved brain imaging, treatment planning software allowing for MRI-CT image fusion and accurate dose calculation, more widely available LINAC-based delivery, and non-invasive immobilization. SRS alone has replaced WBRT as the recommended upfront treatment for NSCLC patients with oligometastases in the brain (47).

Extra-cranially, SBRT is associated with treated tumor control rates rivaling surgery, often in excess of 90%, among NSCLC patients when escalated to a biologically effective dose (BED) of at least 100 Gy (6, 48). As these treatments deliver very high, potentially ablative, doses of radiation to tumor, often near critical normal organs (i.e., spinal cord, kidney, bowel, and heart), the safe delivery of HIGRT is highly dependent on effective patient immobilization, accurate and reproducible image-guided setup, and respiratory motion analysis and management. As the technical expertise and availability of equipment required to deliver HIGRT rapidly expands, there are a growing number of institutions reporting their experiences, both retrospective and prospective, using these techniques in advanced stage NSCLC patients to target metastases located throughout the body (see Table 1).

**Table 1:** Summary of select studies of high dose radiation therapy as part of an aggressive local treatment approach targeting oligometastases from non-small cell lung cancer.

Reference	Study design	Year	Patients	Metastases per patient	Multiple organ involvement	RT technique	Included surgical patients	Included intracranial sites	Definitive thoracic therapy	Systemic therapy	Median follow-up (months)	Median progression-free survival (months)	Overall survival (OS)	Toxicity
Gomez et al. (49)	Randomized phase II prospective	2016	49	≤3	Yes	Various	Yes	Yes	Yes	All received induction chemo	12.39	11.9 (LCT) vs 3.9 (no LCT)	Median OS not reached	20 vs 8.3% G3
Iyengar et al. (50)	Phase II prospective	2014	24	≤6	Yes	SBRT	No	No	NA	All progressed through 1st line chemo, all received erlotinib	11.6	14.7	Median 20.4 months	2 G3 RT-related toxicities
Collen et al. (51)	Phase I prospective	2014	26	≤5	Yes	SBRT	No	Yes	Yes (73%)	65% induction chemo	16.4	11.2	Median 23 months	15% G2 + acute, 8% G3 pulmonary
De Ruysscher et al. (52)	Phase I prospective	2012	39	≤5	No^a^	Various	Yes	Yes	Yes	95% chemo	27.7	12.1	Median 13.5 months	15% G3
Griffioen et al. (53)	Retrospective	2013	61	≤3	No^a^	Various	Yes	Yes	Yes	84% chemo	26.1	6.6	2 years 38%	6.6% G3
Weickhardt et al. (54)	Retrospective	2012	25	≤4	Yes	Various	No^b^	Yes	NA	100% tyrosine kinase inhibitor	9.4	6.2	NA	8% G3
Hasselle et al. (55)	Retrospective	2012	25	≤5	Yes	Stereotactic radiosurgery/SBRT	No	Yes	NA	76% prior to SBRT	14	7.6	1 year 81.1%	8% G3
Jabbour et al. (56)	Retrospective	2011	9	1	No	Conventional RT	No	Yes	Yes	100% chemo	NA	15	Median 28 months	NA
Cheruvu et al. (29)	Retrospective	2011	96	≤8	Yes	SBRT	Yes	Yes	NA	70% chemo	13.5	NA	2 years 25% (oligorecurrence) vs 43% (*de novo* oligometastases)	NA
Yano et al. (57)	Retrospective	2010	44	1	No	Various	Yes	Yes	Yes	16% chemo	NA	NA	Median 74 months	NA
Khan et al. (58)	Retrospective	2006	23	≤2	No	Various	Yes	Yes	Yes	100% upfront chemo	17	12	Median 20 months	17% G3+

*Adapted from Bergsma et al. (23)*.

*^a^A single patient had multiple organ involvement*.

*^b^A single patient underwent surgical ablation*.

*LCT, local consolidative therapy; SBRT, stereotactic body radiotherapy*.

## Treatment of Individual Organ Sites

### Brain

Brain metastases ultimately develop in 30–50% of NSCLC patients and are typically associated with a very poor prognosis. The increased availability of brain MRI as well as improved systemic therapies that improve survival but often poorly penetrate the blood–brain barrier have led to an increase in the number of NSCLC patients ultimately diagnosed with brain metastases (59). Historically, upfront WBRT constituted the standard of care treatment for brain metastases, despite a lack of proven OS benefit, due to its ability to provide improved central nervous system (CNS) control and decrease the risk of neurologic death, at the risk of potential late neurocognitive toxicity, compared to optimal supportive care (OSC). The utility of WBRT has come under significant scrutiny, particularly based on the results of a recently published phase III, non-inferiority, randomized trial from the United Kingdom (UK) (QUARTZ) which compared WBRT to OSC in NSCLC patients with brain metastases unsuitable for surgical resection or SRS. The study authors concluded that although the OSC alone arm did not meet the predetermined primary endpoint of non-inferiority in regard to quality-adjusted life-years, the absolute benefits of WBRT were clinically insignificant and it should not routinely be used to treat this patient population. Proponents of WBRT argue that the patients enrolled on the QUARTZ trial had extremely poor prognosis, evidenced by the reported median OS of 8–9 weeks, which precluded significant benefit from WBRT. In fact, the prognosis for NSCLC patients with brain metastases varies widely and the anticipated benefit of WBRT may be more substantial in patients with a greater ratio of intra- to extra-cranial disease burden who are at high risk of severe mortality and/or mortality with uncontrolled progression of CNS metastases (60). Furthermore, select patients with adequate performance status, limited brain metastases, and low burden extra-cranial disease may experience improved survival from the improved brain control associated with aggressive CNS-directed local therapies including surgery and/or SRS (61). An aforementioned trial demonstrated that in good performance status patients with a single intracranial metastasis, adding surgical resection to WBRT significantly improved OS (44). Similarly, RTOG 9508 studied the impact of SRS boost after WBRT for patients with 1–3 newly diagnosed brain metastases and revealed an OS benefit with the addition of SRS in patients with a single brain metastasis, mean survival time (MST) of 6.5 vs 4.9 months (*p* = 0.039), as well as in NSCLC patients, MST of 5.9 vs 3.9 months (*p* = 0.012) (62).

The benefit afforded by the addition of upfront WBRT to surgical resection and/or SRS has been intensely studied given the potential for prolonged survival in the most favorable subset of NSCLC patients with brain metastases as well as the appreciable risk of late neurocognitive toxicity with WBRT (61). The Alliance group recently published the results of a trial comparing upfront SRS with or without WBRT in patients (approximately two-third NSCLC) with one to three brain metastases and reported no detriment to OS and less cognitive deterioration in the SRS alone group (63, 64). Furthermore, two additional randomized trials each enrolling surgically resected patients with limited brain metastases, one (in which 20% had NSCLC) comparing SRS vs observation (65) and the other (in which 58% had NSCLC) SRS with or without WBRT (66), show adjuvant SRS may provide an optimal balance between maximizing brain metastasis control in the resection cavity and preservation of neurocognition without compromising survival provided patients are followed closely with salvage therapy (either additional SRS or WBRT) initiated at the time of intracranial progression.

An additional consideration in the treatment of brain oligometastases includes the relatively lower prescribed dose, limited by potential toxicity including the risk of radionecrosis, and resulting high local failure rates associated with single fraction SRS for large lesions (>2 cm). Recently published retrospective data show a multifraction SRS (three to five fractions) approach yields increased local control (LC) and decreased risk of radionecrosis in large brain metastases compared to single fraction SRS (67). There are also emerging data supporting the premise that the cumulative volume of intracranial metastatic burden may matter more than brain metastasis number and OS may not be inferior for patients with 4–10 vs 2–3 brain metastases (68). Overall, although the presence of brain metastases has historically felt to be an adverse prognostic factor (24), the development and widespread implementation of SRS has proven to be a powerful tool in the radiation oncologist’s armamentarium for the treatment of oligometastatic NSCLC within the brain.

### Lung

For decades, pulmonary metastasectomy has been utilized for lung metastases from several cancer types, predominantly sarcoma and colorectal cancer. Published experiences confirm that resection can lead to prolonged PFS and OS in selected patients. Pulmonary oligometastases in NSCLC patients have been reported to carry a favorable prognosis that is now reflected in the recently published 8th edition of the TNM classification for lung cancer that separates intra-thoracic metastases, including metastatic lung or pleural nodules, as M1a rather than M1b or M1c (69). The safety and efficacy of SBRT delivered to primary NSCLC tumors and lung oligometastases has been well studied in multiple prospective studies (7, 70). Rusthoven and colleagues reported an actuarial 2-year LC of 96% in a prospective phase II study enrolling patients with one to three lung metastases from various solid tumor primaries (13% from primary lung cancer). Treatment was very well tolerated with only 8% grade 3 events and no grades 4 or 5 toxicity reported. A more recent retrospective study of SBRT for lung oligometastases limited to NSCLC reported 88.9% LC and 74.6% OS at 2 years with no grade 4 pulmonary toxicity, chest pain, or rib fractures (71). Of note, the use of PET/CT along with pathological confirmation (if acceptable risk) can be quite helpful as the presence of a contralateral lung nodule in newly diagnosed advanced NSCLC can be difficult to differentiate between a metastasis and a synchronous lung primary (72). Robust motion management is critical when delivering lung SBRT as metastases may be subjected to significant respiratory-induced motion and resulting target uncertainty which can be minimized by abdominal compression, breath hold, respiratory gating, real-time tumor tracking, and/or generation of an internal target volume based on four-dimensional CT at time of simulation (73).

### Adrenal Glands

Adrenal gland metastases may be present in 5–10% of NSCLC patients (74) at initial presentation and solitary metastases occur in approximately 2–3% of cases. As solitary adrenal masses found on CT may in fact be benign adenomas, further diagnostic workup with PET/CT or dedicated MRI and possible histological confirmation should be pursued (75). Surgery with adrenalectomy of solitary metastases has been reported to provide favorable outcomes in NSCLC patients per several single and multi-institutional series (76) and conventionally fractionated EBRT can be used for palliation of pain with good response rates as measured by analgesic requirements (77). The use of SBRT in the treatment of adrenal oligometastases is gaining traction as an alternative to adrenalectomy resulting in high rates of palliation and LC rates (≥74%) which appear to correlate well with greater BED (78). Definitive radiation for adrenal metastases is an attractive non-invasive alternative particularly given the not infrequent adverse pathological features of positive margins and incomplete resections seen after adrenalectomy. Treatment appears to be well tolerated with only rare severe toxicity (79, 80); however, care must be taken during treatment planning as adrenal metastases may also exhibit significant motion with respiration and are often near the kidney, spinal cord, and sensitive gastrointestinal organs including the liver, colon, stomach, and small bowel.

### Liver

Liver involvement at diagnosis is a relatively uncommon presentation of advanced NSCLC with hepatic metastases reportedly occurring in less than 5% of new diagnoses (81). Histology plays a role in the comparative number of metastases with solitary presentation in 50% of patients with squamous cell carcinoma vs 5% of those with adenocarcinoma. Ultimately, the development of hepatic metastases is not uncommon in the natural history of advanced stage NSCLC. Although there is a wealth of data establishing the benefit of partial hepatectomy for isolated or limited liver metastases from colorectal cancer, the published experience of surgical resection for liver metastases from NSCLC, oligometastatic, or otherwise is lacking. The safety and efficacy of SBRT delivered to hepatic oligometastases from solid tumors has been well established in both retrospective and prospective (82) series with Goodman and colleagues reporting 91% LC at 4 years with only 4.9% grade 3 or greater liver toxicity (83). A major limitation of using these studies in the context of oligometastatic NSCLC is the wide variety of tumor histologies included, with NSCLC comprising a minority of treated cases (21% of patients enrolled on the prospective phase I/II trial by Rusthoven and colleagues); however, a large retrospective study from Moffitt Cancer Center showed that liver metastases of NSCLC origin may exhibit relative radiosensitivity compared to other histologies (84). Contrast (ideally tri-phasic) should be given at simulation to help delineate the target given the similar CT density of metastasis and normal liver. Fiducials may be helpful in aligning to the target for image guidance radiotherapy and motion assessment and management is mandatory due to potential for respiratory-induced tumor motion during treatment (85).

## Other Sites Including Bone, Kidney, Spleen, Skin, and Lymph Nodes

There is relatively little data on management of oligometastatic NSCLC involving these sites with the majority being surgical series for solitary bone (86) or skin (87) lesions. Although not limited to patients with oligometastases, Gerszten and colleagues report a single institutional experience detailing outcomes in 500 cases of spine radiosurgery documenting remarkable 100% long-term radiographic control and 93% long-term pain improvement in a subset of 80 lung cases (88). A recent retrospective study from Mayo Clinic analyzed outcomes after SBRT for non-spine bone oligometastases reporting a 91.8% LC at 1 year and acceptable acute and late toxicities; however, a minority of patients included had NSCLC (89). RTOG 0631 is a randomized phase II/III study of image-guided SRS/SBRT for localized spine metastasis, not limited to NSCLC patients, which has recently closed after adequate accrual with the primary endpoints of feasibility and palliation of pain (NCT00922974). Although the use of SRS or SBRT for bone metastases is promising, more studies are needed evaluating its impact in the context of oligometastatic NSCLC.

## Role of ATT

The potential benefit of aggressive therapy directed to the primary tumor (and involved nodes) has been proven in the setting of multiple histologies of advanced stage cancers. For example, in metastatic renal cell carcinoma, randomized controlled trials have shown the significant OS advantage of cytoreductive nephrectomy added to immunotherapy (90). For advanced NSCLC patients with *de novo* oligometastases or oligorecurrence including initial thoracic disease, unchecked growth of locoregional chest disease may lead to significant tumor-related morbidity including cough, pain, shortness of breath, endobronchial obstruction causing airway collapse or post-obstructive pneumonia, superior vena cava syndrome, and/or severe hemoptysis, which may ultimately result in death. Radiotherapy can alleviate symptoms associated with bulky thoracic disease and is often utilized in the palliative treatment of advanced NSCLC patients.

Historical trials conducted by the RTOG in the 1970s showed that dose escalation up to 60 Gy utilizing conventional fractionation, relative to lower doses, led to improved thoracic tumor control in inoperable NSCLC patients treated with definitive radiotherapy (91). The optimal dose of chest radiotherapy in the setting of oligometastatic NSCLC has been debated given the need to balance palliation, including prevention of morbidity related to thoracic disease progression, while also minimizing treatment toxicity and duration as prolonged breaks in systemic therapy could heighten competing risks of systemic disease progression. A large retrospective study using the National Cancer Database evaluated the comparative effectiveness of chest radiotherapy dose escalation and found a positive association between improved survival and higher-dose radiotherapy (BED above 50 Gy) (92). However, a recent meta-analysis of 14 randomized controlled trials with 3,576 patients concluded there was no strong evidence to support extended fractionation schedules of radiotherapy to palliate thoracic symptoms in incurable NSCLC patients as all patients (including those receiving shorter treatment schedules) appeared to benefit in regard to palliation with no apparent difference in OS (93). Of note, patients with good performance status had longer 1-year OS with more fractions (33.3 vs 25.6%); however, the relative effect was not reported due to a high level of heterogeneity. Acute toxicity was an issue with higher radiotherapy doses though most patients were treated before the era of 3D conformal radiotherapy that has the potential to decrease exposure of normal organs to the full prescribed dose.

Although robust prospective randomized evidence is lacking, Li and colleagues recently published a meta-analysis that included 7 retrospective observational cohort studies and 668 synchronous oligometastatic NSCLC patients, of whom 227 (34.0%) received ATT consisting of surgery and/or radiotherapy to a total dose more than 40 Gy (94). Receipt of ATT was associated with significantly improved OS (HR 0.48, *p* < 0.00001) in the entire cohort, as well as in subgroup analyses of patients with single organ metastases (HR 0.42, *p* < 0.00001), solitary (HR 0.49, 95% CI 0.31–0.75) or two to four brain metastases (HR 0.44, 95% CI 0.26–0.73), and patients with thoracic stage I–II (HR 0.38, *p* = 0.004) or stage III (HR 0.32, *p* = 0.01) disease. Pooled cumulative OS at 3 years was significantly higher in the ATT group (23.0 vs 3.7%). A recent prospective phase II study by Li and colleagues evaluating the efficacy and toxicity of definitive thoracic concurrent chemoradiation (BED ≥ 60 Gy) followed by consolidation chemotherapy for oligometastatic NSCLC (≤5 metastases) enrolled 64 patients yielding encouraging 14-month median PFS and 26-month median OS at a median follow-up of 28 months (95). These prospectively accrued data are consistent with PFS and OS outcomes reported in other retrospective studies of ATT in oligometastatic NSCLC (25, 96, 97). While most published studies employed conventionally fractionated radiotherapy schedules with sequential or concurrent chemotherapy, HIGRT has also been used as definitive local treatment of smaller primary lung tumors in the oligometastatic setting (51, 53). Whether treatment is surgical or radiotherapy based, the use of ATT for controlling presenting or potential symptoms of thoracic disease is an integral component of an aggressive treatment approach for oligometastatic NSCLC patients with synchronous presentation given the potential for prolonged survival and significant morbidity and/or mortality resulting from uncontrolled locoregional progression.

## Use of Radiotherapy with Systemic Therapy

Systemic therapy is the standard palliative treatment option for reasonably fit NSCLC patients presenting with either synchronous or metachronous disseminated disease with the agent (chemotherapy, TKIs, or immunotherapy with checkpoint inhibitors) selected based on histological and genotypic information about the primary and/or metastatic tumor. Randomized trials supporting the use of these systemic therapies typically included patients with widespread, rather than limited, metastases. Regardless, the promise of improved systemic control only heightens the importance of effective local treatment modalities to address isolated persistent or progressive metastases. For example, oligoprogression is well documented during treatment of onco-addicted NSCLC (ALK gene rearrangements or EGFR mutations) with TKIs such as crizotinib for ALK+ and erlotinib for EGFR mutated NSCLC. In these patients, local ablative treatment with HIGRT has allowed continuation of targeted therapy with greater than 6 months of additional disease control (50, 54).

The optimal integration of definitive local therapy with systemic therapy in oligometastatic NSCLC is not yet certain. It is common clinical practice to address limited brain metastases with upfront SRS or surgery (if symptomatic or warranted for diagnosis) followed by SRS or WBRT with initiation of systemic therapy or definitive thoracic therapy (if brain only metastases and synchronous presentation); however, medical oncologists typically treat patients with extra-cranial oligometastases with upfront systemic therapy. As alluded to earlier, the use of induction systemic therapy may allow for selection of patients who are less likely to develop new metastases and may experience improved PFS after consolidation with aggressive local therapy to the chest and limited residual metastases (typically ≤3) (49). The selective use of aggressive metastasis-directed and thoracic therapy in non-progressing patients is a potential source of selection bias in published retrospective studies, namely immortal time bias, which is best controlled for with a randomized controlled study. The recently reported multicenter, phase II randomized controlled trial by Gomez and colleagues utilized local consolidative therapy (LCT) of all active disease and reported a dramatic median PFS benefit (11.9 vs 3.9 months, *p* = 0.0054) compared to maintenance treatment in oligometastatic NSCLC patients (46 of 49 *de novo*) with three or fewer metastatic disease lesions without progression after first-line systemic therapy (49). These randomized prospective data reinforce the sum of available retrospective evidence signaling the significant PFS benefit of adding aggressive local therapy to standard systemic treatment for patients with NSCLC oligometastases.

It should be noted that the administration of systemic therapy may be associated with significant acute toxicities, including chemotherapy-related nausea and myelosuppression and autoimmune phenomena related to immunotherapy, which adversely affect patient quality of life. In the setting of oligometastatic NSCLC, Collen and colleagues reported the results of a small prospective study that showed receipt of induction chemotherapy prior to undergoing SBRT was not prognostic for LC or PFS (51). It is possible that select patients may experience prolonged PFS with aggressive local therapy directed at all metastatic sites in lieu of systemic therapy; however, it is reasonable to consider chemotherapy, or other appropriate systemic agents, as upfront treatment in oligometastatic patients given the OS benefit afforded in both early (adjuvant after resection in node positive and/or larger primary tumors) and advanced stage NSCLC (98). This treatment approach is reflected in the NCCN guidelines version 1.2017. As mentioned earlier, the use of aggressive local therapy in the setting of oncogene-addicted NSCLC is an area of significant interest as studies have reported excellent PFS and OS with the addition of SBRT to erlonitib in patients progressing after first-line platinum-based chemotherapy (50). Salvage of oligoprogression in the setting of advanced NSCLC with a driver mutation may allow continuation of otherwise efficacious and well-tolerated systemic therapy in patient who may not have other effective treatment options (99). Reported toxicities of the above approaches have been quite low with rare reports of severe (grades 4–5) adverse events.

Although the benefit of aggressive primary and metastasis-directed local therapy in the metastatic setting has commonly felt to be due to improved LC of targeted gross tumor(s), Gomez and colleagues reported a significantly prolonged time interval to the appearance of a new lesion among patients randomized to LCT (11.9 vs 5.7 months) (49). This is a provocative finding that suggests an aggressive local treatment approach may alter the natural history of metastatic disease either by limiting the potential for later spread or stimulating systemic immune-surveillance. Furthermore, there are fascinating reports of radiation inducing an “abscopal effect” whereby treating a single lesion results in regression of metastases far away from the treated site, however, these remain mostly anecdotal at present. Preclinical studies show synergistic antitumor effects with radiotherapy *via* pro-immunogenic properties resulting from increased tumor antigen presentation and activation of cytotoxic T cells (100) and emerging clinical data also support this concept with a recent study reporting previous treatment with extra-cranial radiotherapy was associated with significantly improved median OS (11.6 vs 5.3 months, *p* = 0.034) among patients treated with pembrolizumab on the KEYNOTE-001 phase I trial (101). Importantly, predominantly retrospective data to date suggest that the contemporaneous administration of immunotherapy and intracranial SRS or palliative dose extra-cranial radiotherapy is relatively safe without dramatically increased risk of synergistic toxicity; however, efficacy nor the safety of more aggressive extra-cranial dose schedules has not been studied (102, 103). There may even be a detrimental effect of more protracted palliative radiotherapy schedules given the extreme radiosensitivity of circulating lymphocytes and our growing understand of the importance of the immune system in combatting metastatic disease (104).

## Future Directions and Ongoing Prospective Trials

The emerging evidence supports the existence of a subset of advanced NSCLC patients who will benefit from definitive local treatment to limited sites of disease with unparalleled PFS and OS. The challenge has been defining the appropriate patient population and proving the added benefit of aggressive local therapy in a randomized fashion. The phase II study by Gomez and colleagues represents the first randomized controlled trial addressing the question at hand and supports the premise that select advanced NSCLC patients may progress predominantly in known disease sites and aggressive thoracic and metastasis-directed local therapy can result in improved PFS. As follow-up remains short (median of 12.4 months), it is unclear whether the PFS benefit observed will translate into improved OS. It is possible that crossover of patients in the maintenance arm to LCT after progression could minimize the potential OS benefit similar to that seen in randomized trials of targeted agents in NSCLC (105). In addition, as the study was powered to assess the primary outcome, PFS, and was closed early at the recommendation of the data safety monitoring committee due to an overwhelming probability of concluding in favor of the LCT group, it may be insufficiency powered to measure a true difference in OS between arms. Regardless of whether an improvement in OS is ultimately shown, an improvement in PFS is certainly meaningful as a prolonged disease-free interval off of systemic therapy may represent a significant quality of life benefit to the patient.

Further randomized studies are necessary. NRG Oncology has recently opened NRG-LU002 (NCT03137771), a randomized phase II/III trial enrolling NSCLC patients with ≤3 oligometastatic sites that will build upon the experience of Gomez and colleagues and seeks to evaluate the PFS and OS benefit, if it exists, of consolidative SBRT and definitive thoracic therapy after first-line/induction systemic therapy in a national cooperative group setting. SABR-COMET (NCT01446744) is another multi-institutional randomized phase II trial that has completed accrual of 99 patients (not limited to NSCLC histology) with ≤5 metastases and a controlled primary tumor. Patients were randomized to standard of care with or without SBRT consolidation to all sites of known disease with OS as the primary outcome measure. SARON (NCT02417662) is a UK-based multicenter randomized phase III study enrolling (target of 340) patients with oligometastatic NSCLC and examining the feasibility, safety, and efficacy of consolidation with SBRT or conventional RT to primary and sites of metastases after standard platinum-based doublet chemotherapy. CORE (NCT02759783) is another randomized phase II trial (anticipated accrual of 206 patients) that has opened in the UK enrolling breast, prostate, and NSCLC patients with oligorecurrence and is evaluating the impact of adding SBRT to standard of care with PFS as the primary outcome. These larger randomized studies should increase the power to uncover an OS benefit with the addition of SBRT as comprehensive local therapy in the setting of oligometastatic NSCLC.

Additional questions remain unanswered beyond the measurable added benefit, if any, of an aggressive treatment approach including definitive local therapy. Both SABR-COMET and the randomized phase II study by Gomez and colleagues were evaluating the use of definitive local therapy to sites of limited disease as consolidation after upfront systemic therapy. However, the optimal timing of aggressive local treatment remains undefined. The ongoing Chinese OITROLC trial (NCT02076477) may help answer this question as it randomizes oligometastatic patients (≤5 distant organ metastases) between upfront definitive local therapy to the primary and all sites of metastases vs a consolidative approach after two cycles of induction chemotherapy with 3-month response rate as the primary outcome and 3-year PFS and toxicity as secondary outcomes. As use of SBRT combined with erlotinib showed remarkable outcomes in oligoprogressive metastatic NSCLC (50), a pilot study from Memorial Sloan Kettering Cancer Center (NCT02450591) is now evaluating outcomes when SBRT or surgery is added to erlotinib for newly diagnosed oligometastatic lung adenocarcinoma harboring a sensitizing EGFR mutation with the goal to evaluate feasibility and PFS.

Although crucial before more broadly adopting an aggressive local therapy approach to oligometastatic disease, the safety and optimal dose fractionation when treating multiple oligometastases in various organ sites remains unknown as the bulk of published literature studied the use of SBRT for single metastases within individual organs (8, 106). NRG BR001 (NCT02206334), a phase I study enrolling patients with oligometastatic NSCLC, prostate, or breast cancer, attempts to clarify the tolerability of SBRT when treating patients with multiple metastases at pre-defined doses in seven organ sites including bone and lymph nodes, where little safety data currently exist.

The era of personalized medicine has arrived in the field of oncology, ushered in by advances in imaging and molecular biology. Broad molecular profiling is expected to be a key component of future advancements in the care of patients with NSCLC. Prospective clinical trials are underway to generate clinical and molecular predictors, including comprehensive molecular profiling and/or primary tumor microRNA expression, to guide selection of patients for oligometastases-directed ablative therapy (107, 108). Further investigation, including independent validation, is needed before clinical implementation. Given the transition toward earlier incorporation of immunotherapy, such as the upfront administration of pembrolizumab in some newly diagnosed advanced NSCLC patients, there is considerable interest in combining immunotherapy and radiotherapy to improve outcomes and perhaps even induce the abscopal effect (109). A web search of http://clinicaltrials.gov revealed that there are now at least 14 actively recruiting studies evaluating immunotherapy in NSCLC as of March 31, 2017. These studies will hopefully add knowledge as to the added benefit and optimal incorporation of radiation with immunotherapy, including the most appropriate timing, sequencing, and dosing of each.

Despite the emerging evidence supporting the use of aggressive local treatments in addition to standard of care systemic therapy for oligometastatic NSCLC, as well as the increasing availability of non-invasive potentially ablative radiotherapy techniques, there are practical limitations that must be considered as our society increasingly recognizes the rising costs of health care in the modern era and begins to transition toward a value-based reimbursement model for providers and hospital systems (110). “Payers,” including governmental and private health insurers, are increasingly emphasizing an evidence-based approach to justify potentially costly treatments in patients with relatively poor prognosis. This can make obtaining insurance approval for novel and/or investigational uses for expensive treatment modalities, including SRS or SBRT, an onerous challenge for the treating radiation oncology team, as well as increase the financial burden and stress patients and their families experience during cancer treatment (111). This new reality reinforces the need for high level evidence to justify and guide the recommendation for aggressive local treatments in the setting of oligometastatic NSCLC.

## Conclusion

Tremendous developments in the field of oncology within the past decade, including improvements in imaging and radiotherapy technique allowing for the safe delivery of potentially ablative doses of radiation with minimal toxicity or interruption in quality of life or systemic therapy, have ushered in the next frontier of NSCLC treatment. A steadily increasing number of published retrospective and prospective clinical experiences, including the first successfully completed randomized trial, support the concept that NSCLC patients with limited metastatic disease, termed as oligometastases, will experience improved outcomes with aggressive local treatment with surgery and/or radiation therapy targeting all sites of appreciable disease. The challenge for the oncology community moving forward is to design and accrue to prospective randomized controlled trials that will allow for an accurate assessment of the added benefit of aggressive local therapy as well as the optimal integration with existing and emerging systemic therapies.

## Author Contributions

DB wrote the initial draft of this review, with edits and revisions from each of the other authors: JS, DS, SC, and MM.

## Conflict of Interest Statement

The authors declare that the research was conducted in the absence of any commercial or financial relationships that could be construed as a potential conflict of interest.
